# Valsalva retinopathy induced by handstand: a case report

**DOI:** 10.1186/s12886-020-01638-z

**Published:** 2020-09-14

**Authors:** Toshiya Miyaki, Teruyo Kida, Shou Oosuka, Masanori Fukumoto, Takaki Sato, Masayuki Nakajima, Tsunehiko Ikeda

**Affiliations:** 1 Department of Ophthalmology, Osaka Medical College, 2-7 Daigaku-machi, Takatsuki City, Osaka, 569-8686 Japan; 2 Nakajima Eye Clinic, Kyoto-City, Osaka, Japan

**Keywords:** Valsalva retinopathy, Handstand, Vitreous hemorrhage (VH), Sub-internal limiting membrane (sub-ILM) hemorrhage

## Abstract

**Background:**

Valsalva retinopathy is known to occur as a sudden preretinal or sub-internal limiting membrane hemorrhage induced by a rapid rise in venous pressure following increased intrathoracic or intraabdominal pressure. Here we report a case of Valsalva retinopathy that was probably induced by straining that occurred due to following a handstand.

**Case presentation:**

A 15-year-old boy became aware of decreased visual acuity in his left eye immediately after doing a handstand for approximately 10 s during physical education class, and subsequently visited a local clinic on the same day. Upon examination, a vitreous hemorrhage (VH) in the posterior pole of the fundus was found in his left eye, and he was subsequently referred to our department 7 days later. Upon examination, the VH around the optic nerve head of the left eye appeared to be resolved, and an oval-shaped sub-internal limiting membrane (sub-ILM) hemorrhage was found in the superonasal side of the optic nerve head. No abnormalities were observed in the macular area. Four months later, the sub-ILM hemorrhage was found to have spontaneously resolved. Subsequent fluorescein angiography examinations revealed no abnormal findings at the lesion site.

**Conclusions:**

In this patient, we hypothesize that the Valsalva retinopathy was induced by straining that occurred due to a handstand, and that the resultant sub-ILM hemorrhage progressed to VH.

## Background

Valsalva retinopathy was first reported by Duane et al. in 1972 [1], and it is known to occur as a sudden preretinal hemorrhage induced by a rapid rise in venous pressure following an increased intrathoracic or intraabdominal pressure due to a Valsalva-type maneuver, such as a cough, vomiting, or a physical strain. In cases of Valsalva retinopathy, a sub-internal limiting membrane (sub-ILM) hemorrhage or preretinal hemorrhage often occurs in the posterior pole or around the optic nerve head. Moreover, an intraretinal, subretinal, or vitreous hemorrhage (VH) can also occur [2]. It has previously been reported that Valsalva retinopathy can occur due to a variety of causes, such as vomiting, the lifting of a heavy object, the physical strain that occurs during child birth [3], and general anesthesia [4], yet there are very few reports of Valsalva retinopathy occurring due to a handstand [5]. Here we report a rare case of Valsalva retinopathy that was probably induced by straining that occurred following a handstand.

## Case presentation

**Fig. 1:**
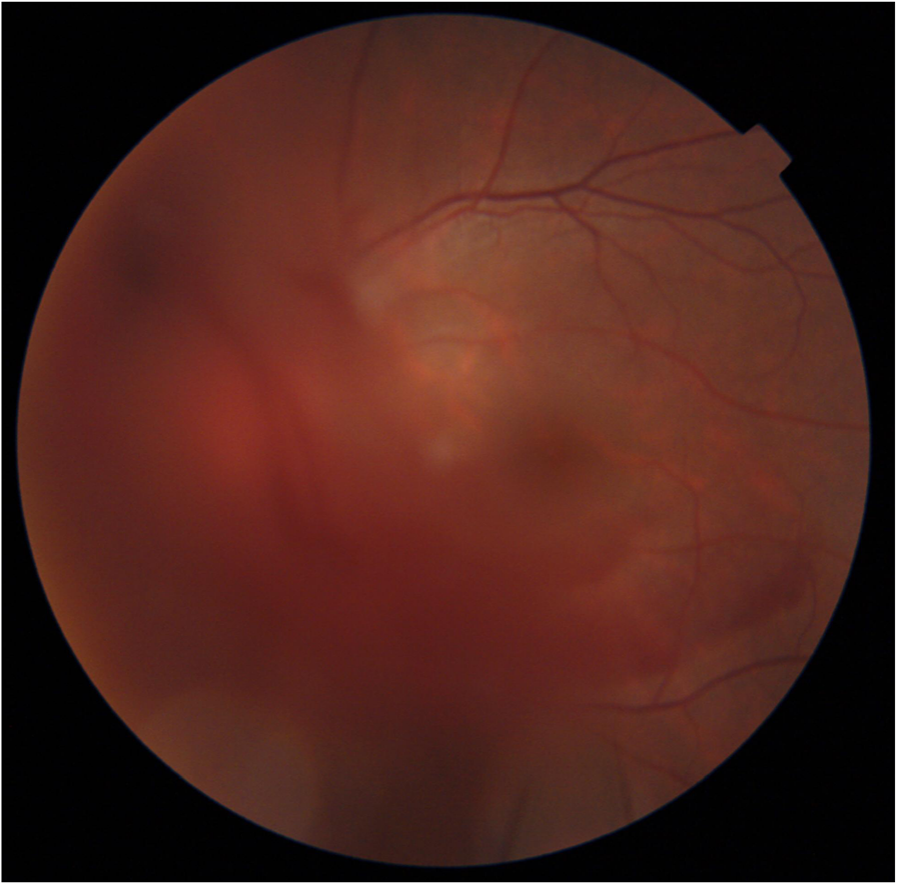
Fundus photograph obtained at initial presentation to a local ophthalmologist showing vitreous hemorrhage at the posterior pole of the patient’s left eye

**Fig. 2:**
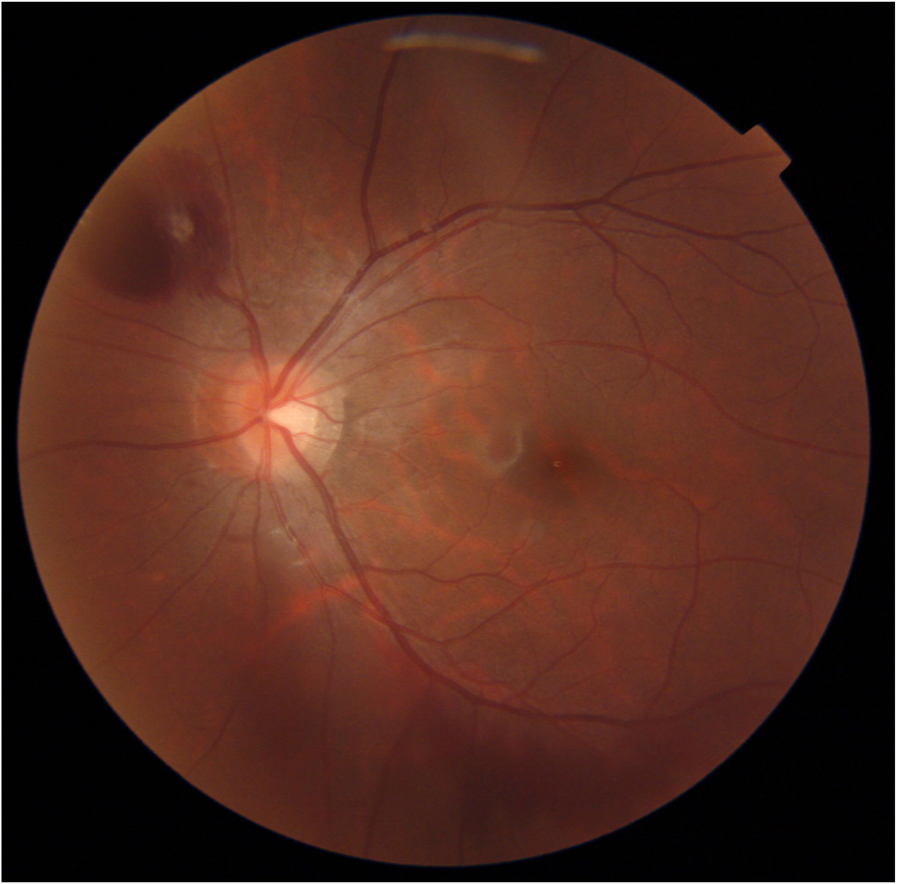
Fundus photograph obtained at initial presentation to our department showing an oval-shaped sub-internal limiting membrane (sub-ILM) hemorrhage on the superonasal side of the optic nerve head

**Fig. 3:**
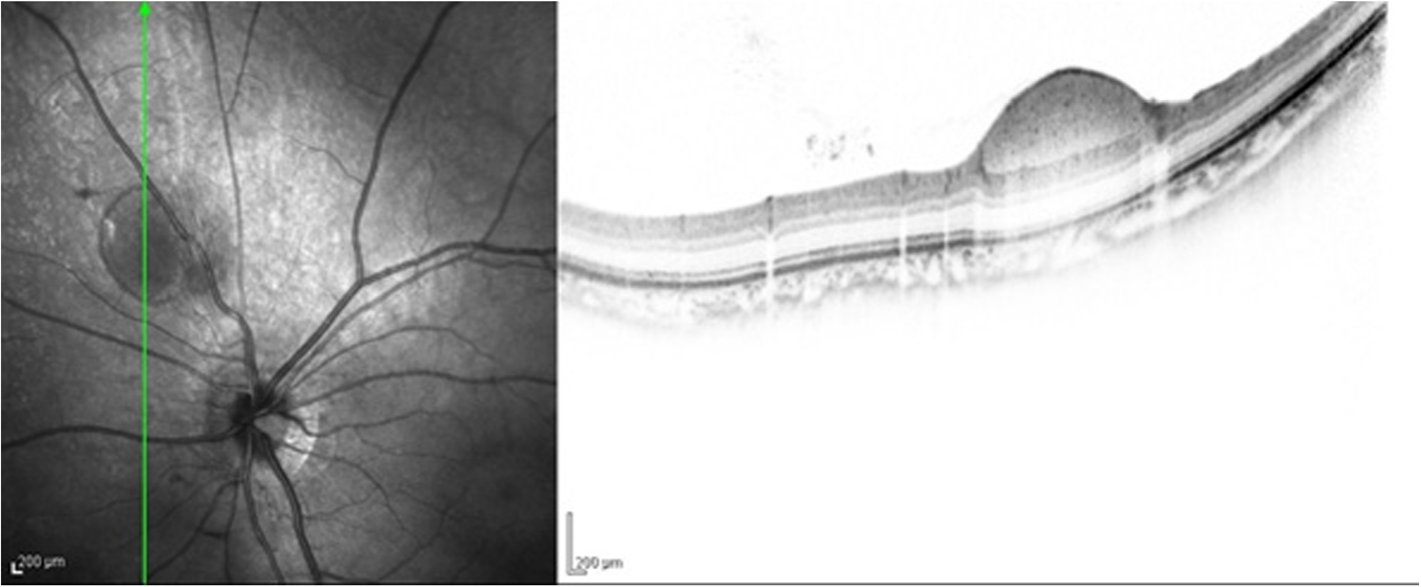
Optical coherence tomography (OCT) imaging obtained at initial presentation to our department showing a sub-ILM hemorrhage at the bleeding site of the superonasal side

**Fig. 4:**
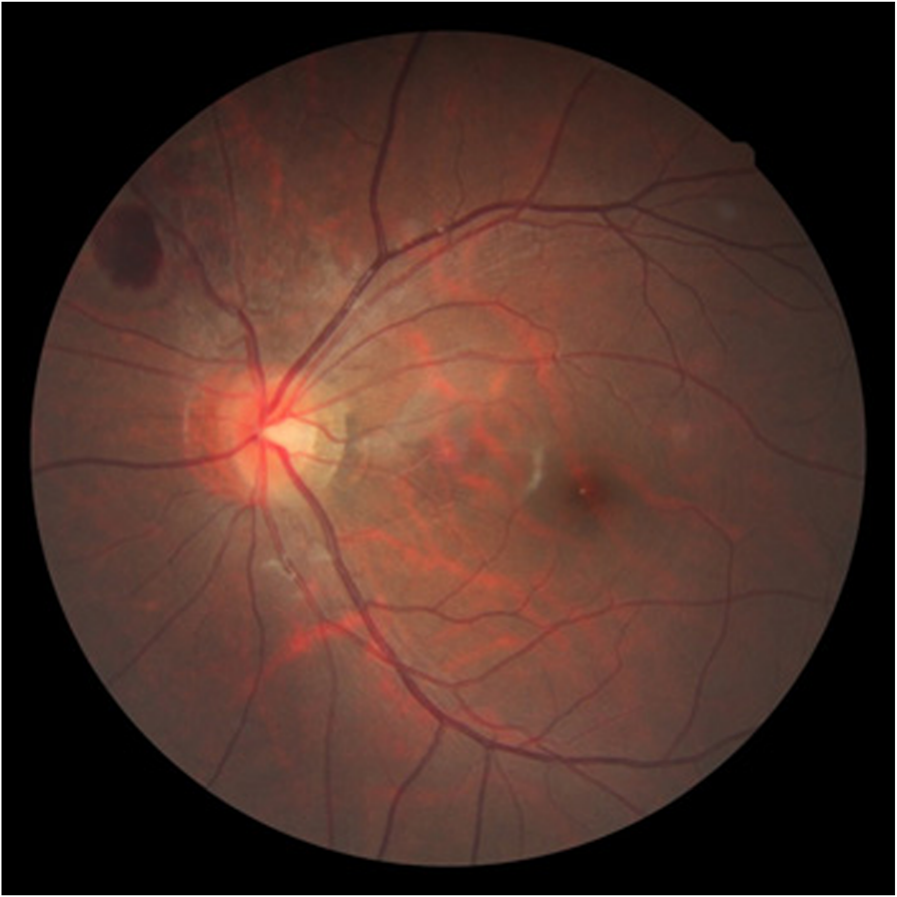
Fundus photograph obtained at 1-month post onset of Valsalva retinopathy showing that the sub-ILM hemorrhage had decreased in size

**Fig. 5:**
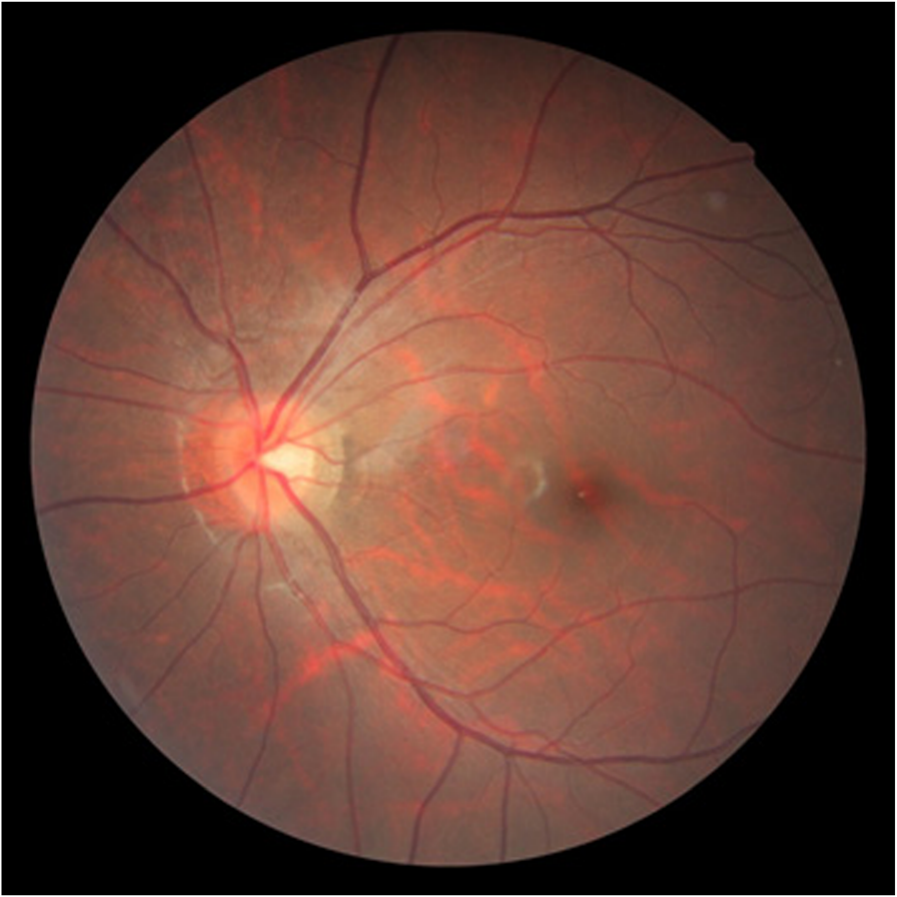
Fundus photograph obtained at 4-months post onset of Valsalva retinopathy showing that the sub-ILM hemorrhage had completely resolved

**Fig. 6:**
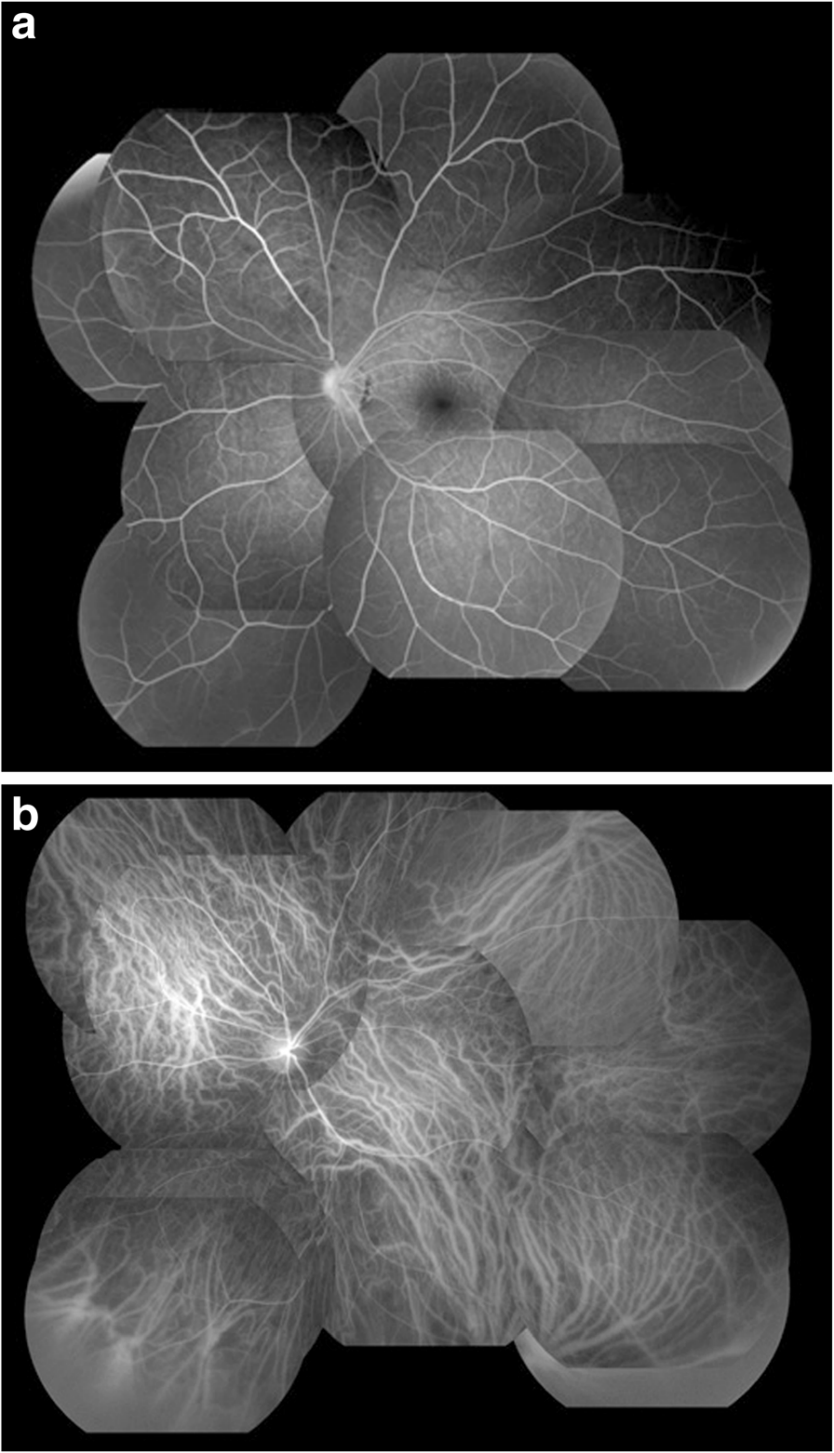
Fluorescein (**a**) and indocyanine green (**b**) angiography fundus photographs obtained at 4-months post onset of Valsalva retinopathy showing no obvious retinal vascular abnormality or abnormal choroidal blood vessels

A 15-year-old boy suddenly noticed floaters in his left eye after doing a 10-s handstand in his high-school physical education class, and presented later that same day at a nearby ophthalmologist for examination. Initial examination revealed VH in the posterior pole of his left eye (Fig. 1), and the patient was referred to our hospital for a more detailed examination. An examination performed, 7 days later revealed no remarkable medical or family history, or history of trauma, and that the patient’s best-corrected visual acuity (BCVA) was 20/20 [− 4.50/− 1.25 Ax170°; uncorrected visual acuity (VA) 20/300] OD and 20/40 (− 4.25/− 1.25 Ax170°; uncorrected VA 20/300) OS, and the intraocular pressure (IOP) in his right eye and left eye was 19 mmHg and 16 mmHg, respectively. Although both eyes were myopic, no marked abnormalities were observed in the patient’s right-eye anterior segment, optical media, or fundus. Although the VH in his left eye resolved, an oval-shaped sub-ILM hemorrhage was found on the superonasal side of the optic nerve head (Fig. 2). An optical coherence tomography (OCT) examination revealed no abnormal findings in the macular region of retina, yet a sub-ILM hemorrhage was observed at the area of the bleeding site at the superonasal side (Fig. 3). A follow-up examination performed 1-month later revealed that the sub-ILM hemorrhage had decreased in size (Fig. 4), and that his VA had improved to a BCVA of 20/20 (− 4.75/− 1.25D Ax125°; uncorrected VA 20/400). At 4-months post onset, the sub-ILM hemorrhage was found to have completely resolved (Fig. 5). For a more detailed examination of the bleeding site, fluorescein and indocyanine green angiography examinations were performed, yet no obvious retinal or vascular abnormalities or abnormal choroidal blood vessels were observed (Fig. 6a,b). After that, the clinical course remained favorable and no abnormalities in VA (20/20), IOP, anterior optical media, or fundus were observed.

## Discussion and conclusion

It has previously been reported that Valsalva retinopathy can occur due to a variety of physical movements [6–16], such as bungee jumping [6–11], weight lifting [12, 13], push-ups [14, 15], and aerobics [16]. However, and to the best of our knowledge, there had only been one published report of Valsalva retinopathy caused by performing a handstand [5]. In that study, the authors reported a 32-year-old man who experienced a sudden drop in VA after repeated ‘handstand push-ups’ during gym training, and their fundoscopy findings revealed extensive preretinal hemorrhage, including at the macular region, and several intraretinal hemorrhages. In that patient, the corrected VA was reduced to 20/60, which led to a diagnosis of a preretinal hemorrhage due to Valsalva retinopathy. The patient was followed-up conservatively, and the hemorrhage spontaneously resolved with a favorable prognosis.

Bungee jumping is a well-known physical activity that involves jumping from a high location with an elastic safety cord attached to one leg while rapidly descending in an “upside-down” (i.e. handstand) position. When performing the activity, the jumpers often hold their breath during the rapid free-fall descent, which increases the intrathoracic pressure. The rapid deceleration that occurs at the end of the fall can rapidly increase the venous pressure in the upper body, which can cause rupture of the retinal blood vessels that subsequently leads to Valsalva retinopathy [7]. Furthermore, since the brain is located below the heart when in the upside-down handstand position, it is assumed that the venous pressure in the head may temporarily increase due to the effect of gravity. Thus, we speculate that in our present case, the Valsalva retinopathy, occurred due to a gravity effect, similar to that reported in the previous study, and may be common.

It should be noted that there are some differences between the previously reported bungee-jumping Valsalva retinopathy cases. For example, Chan et al. [10] reported that the Valsalva retinopathy in their patient occurred due to change in the hydrostatic pressure of the ciliary body and retinal circulation due to the change in gravity when the subject descended. However, in a study by Innocenti et al. [11], the authors reported that the possible primary cause of the Valsalva retinopathy in their patient was an increase of venous pressure due to increased abdominal muscle tension that impaired the venous return when the subject held a breath during the rapid descent. This hypothesis is applicable to Valsalva retinopathy caused by a handstand.

In order to identify the specific causes of a sub-ILM hemorrhage, the presence or absence of underlying diseases (e.g., diabetes mellitus, hypertension, and hematological disease) and trauma, as well as fundus findings in the fellow eye, may be helpful. In addition, abnormal retinal vessels, such as congenital vascular loops, and choroidal neovascularization reportedly can be the causes of the hemorrhage [17]. Thus, in our present case, fluorescein and indocyanine green angiography examinations were performed after the hemorrhage was resolved, and our findings revealed no obvious retinal or choroidal vascular abnormalities at the site of the bleeding site. Moreover, our OCT imaging findings revealed a persistent ILM and sub-ILM cavity [18].

In many Valsalva retinopathy cases, spontaneous resolution can be expected when there is only a small amount of bleeding. However, when the amount of bleeding is large, thus delaying absorption of the blood, treatments such as ILM incision by neodymium-doped yttrium aluminum garnet (Nd: YAG) laser [19], and vitreous surgery [20] should be considered. For the case in this present study, we selected a conservative treatment since the VH was spontaneously resolved at the early stage and because the sub-ILM hemorrhage was expected to be spontaneously absorbed, as they were in the superonasal side of the optic disc and the nasal margin of the disc may have been elevated.

In this present case, i.e., a previously healthy young man with no underlying disease, we hypothesize that the Valsalva retinopathy probably occurred due to performing a handstand, and favorable improvement of VA was achieved via a conservative treatment. Although there have been only a few reports of VH caused by Valsalva retinopathy occurring due to a handstand, careful attention should be paid, as Valsalva retinopathy can occur in eyes in which not blood-vessels abnormalities are observed in the fundus.

## Data Availability

The datasets during the current study are available from the corresponding author on reasonable request.

## Abbreviations

VH: Vitreous hemorrhage
ILM: Internal limiting membrane
OCT: Optical coherence tomography
IOP: Intraocular pressure
BCVA: Best-corrected visual acuity
